# Advances in the diagnosis and treatment of sickle cell disease

**DOI:** 10.1186/s13045-022-01237-z

**Published:** 2022-03-03

**Authors:** A. M. Brandow, R. I. Liem

**Affiliations:** 1 Department of Pediatrics, Section of Pediatric Hematology/Oncology/Bone Marrow Transplantation, Medical College of Wisconsin, Milwaukee, WI USA; 2 Division of Hematology, Oncology and Stem Cell Transplantation, Ann and Robert H. Lurie Children’s Hospital of Chicago, Chicago, IL USA

**Keywords:** Sickle cell disease, Sickle cell anemia, Hemoglobin

## Abstract

Sickle cell disease (SCD), which affects approximately 100,000 individuals in the USA and more than 3 million worldwide, is caused by mutations in the βb globin gene that result in sickle hemoglobin production. Sickle hemoglobin polymerization leads to red blood cell sickling, chronic hemolysis and vaso-occlusion. Acute and chronic pain as well as end-organ damage occur throughout the lifespan of individuals living with SCD resulting in significant disease morbidity and a median life expectancy of 43 years in the USA. In this review, we discuss advances in the diagnosis and management of four major complications: acute and chronic pain, cardiopulmonary disease, central nervous system disease and kidney disease. We also discuss advances in disease-modifying and curative therapeutic options for SCD. The recent availability of l-glutamine, crizanlizumab and voxelotor provides an alternative or supplement to hydroxyurea, which remains the mainstay for disease-modifying therapy. Five-year event-free and overall survival rates remain high for individuals with SCD undergoing allogeneic hematopoietic stem cell transplant using matched sibling donors. However, newer approaches to graft-versus-host (GVHD) prophylaxis and the incorporation of post-transplant cyclophosphamide have improved engraftment rates, reduced GVHD and have allowed for alternative donors for individuals without an HLA-matched sibling. Despite progress in the field, additional longitudinal studies, clinical trials as well as dissemination and implementation studies are needed to optimize outcomes in SCD.

## Introduction

Sickle cell disease (SCD), a group of inherited hemoglobinopathies characterized by mutations that affect the β-globin chain of hemoglobin, affects approximately 100,000 people in the USA and more than 3 million people worldwide [1, 2]. SCD is characterized by chronic hemolytic anemia, severe acute and chronic pain as well as end-organ damage that occurs across the lifespan. SCD is associated with premature mortality with a median age of death of 43 years (IQR 31.5–55 years) [3]. Treatment requires early diagnosis, prevention of complications and management of end-organ damage. In this review, we discuss recent advances in the diagnosis and management of four major complications in SCD: acute and chronic pain, cardiopulmonary disease, central nervous system disease and kidney disease. Updates in disease-modifying and curative therapies for SCD are also discussed.

### Molecular basis and pathophysiology

**Fig. 1:**
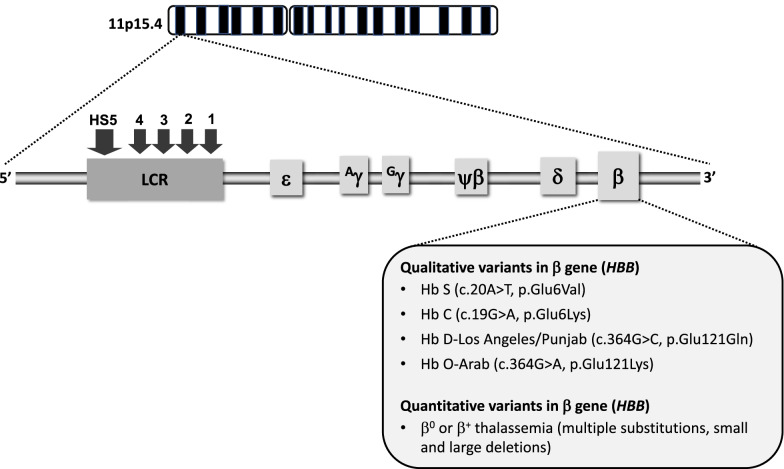
Genetic and molecular basis of sickle cell disease. SCD is caused by mutations in the β globin gene, located on the β globin locus found on the short arm of chromosome 11. The homozygous inheritance of Hb S or co-inheritance of Hb S with the β^0^ thalassemia mutation results in the most common forms of severe SCD. Co-inheritance of Hb S with other variants such as Hb C, Hb D-Los Angeles/Punjab, Hb O-Arab or β^+^ thalassemia also leads to clinically significant sickling syndromes (LCR, locus control region; HS, hypersensitivity site)

Hemoglobin S (HbS) results from the replacement of glutamic acid by valine in the sixth position of the β-globin chain of hemoglobin (Fig. 1). Severe forms of SCD include hemoglobin SS due to homozygous inheritance of HbS and S/β^0^ thalassemia due to co-inheritance of HbS with the β^0^ thalassemia mutation. Other forms include co-inheritance of HbS with other β-globin gene mutations such as hemoglobin C, hemoglobin D-Los Angeles/Punjab or β^+^ thalassemia. Hb S has reduced solubility and increased polymerization, which cause red blood cell sickling, hemolysis and vaso-occlusion (Table 1) that subsequently lead to pain episodes and end-organ damage such as cardiopulmonary, cerebrovascular and kidney disease (Table 2).

**Table 1:** Sickle cell disease

Epidemiology	SCD affects primarily individuals of African or Afro-Caribbean descent 1 in 12 individuals are carriers for sickle cell trait 1 in 365 Black infants in the US are affected by SCD Approximately 100,000 individuals in the US and millions more worldwide have SCD
Molecular basis and pathophysiology	Mutation is caused by single nucleotide substitution in the 6th codon of β-globin gene (*HBB*) Mutation results in production of sickle hemoglobin Common genotypes are homozygous SS disease (HbSS) and the compound heterozygous states HbSC, HbS/β^0^ and HbS/β^+^ thalassemia Mutation leads to reduced solubility of sickle hemoglobin and increased polymerization Pathophysiological contributors include red blood cell sickling, hemolysis, vaso-occlusion, cell adhesion, pro-inflammatory state, oxidative injury, endothelial dysfunction and hypercoagulability
Major complications and disease burden	Acute pain and chronic pain syndrome Functional asplenia and infection Splenic sequestration Acute chest syndrome Cerebrovascular disease and stroke Neurocognitive deficits Retinopathy Priapism Chronic lung disease Pulmonary hypertension Skin ulcers Osteonecrosis Chronic kidney disease

**Table 2:** Prevalence and pathophysiologic basis of major complications in SCD

	Prevalence	Pathophysiology and/or risk factors
Acute and chronic pain	Acute pain—represents 70% of acute care visits Chronic pain—30% in adults, 40% in children	Tissue ischemia and infarction Ischemia–reperfusion injury Hemolysis-induced endothelial dysfunction Inflammation and oxidative stress Peripheral and central nervous system sensitization Identifiable causes such as avascular necrosis and leg ulcers
Pulmonary hypertension	10% in adults by right-heart catheterization	Intravascular hemolysis Nitric oxide depletion Chronic hypoxia Diastolic dysfunction Diffuse myocardial fibrosis
Chronic lung disease	Obstructive lung disease—16% children, 8% adults Restrictive lung disease—7% children, 28% adults	Obstructive lung disease—atopy, airway inflammation (↑ leukotrienes) Restrictive lung disease—recurrent acute chest syndrome
Stroke	Overt stroke—11% by 20 years old Silent cerebral infarct—39% by 18 years old	Cerebral vasculopathy ↓ Blood oxygen content, ↑ cerebral blood flow and ↑ oxygen extraction Nocturnal hypoxemia Moyamoya
Sickle nephropathy	Chronic kidney disease—20 to 40% of adults	Medullary hypoperfusion and ischemia Glomerular hemodynamic alterations Hemolysis-induced oxidative injury Endothelial damage Vascular congestion Hypoxia-inducible factor-1α dependent injury

### Acute and chronic pain

Severe intermittent acute pain is the most common SCD complication and accounts for over 70% of acute care visits for individuals with SCD [4]. Chronic daily pain increases with older age, occurring in 30–40% of adolescents and adults with SCD [5, 6]. Acute pain is largely related to vaso-occlusion of sickled red blood cells with ischemia–reperfusion injury and tissue infarction and presents in one isolated anatomic location (e.g., arm, leg, back) or multiple locations. Chronic pain can be caused by sensitization of the central and/or peripheral nervous system and is often diffuse with neuropathic pain features [7, 8]. A consensus definition for chronic pain includes “Reports of ongoing pain on most days over the past 6 months either in a single location or multiple locations” [9]. Disease complications such as avascular necrosis (hip, shoulder) and leg ulcers also cause chronic pain [9].

#### Diagnosis of acute and chronic pain

The gold standard for pain assessment and diagnosis is patient self-report. There are no reliable diagnostic tests to confirm the presence of acute or chronic pain in individuals with SCD except when there are identifiable causes like avascular necrosis on imaging or leg ulcers on exam. The effects of pain on individuals’ function are assessed using patient-reported outcome measures (PROs) that determine to what extent pain interferes with individuals’ daily function. Tools shown to be valid, reliable and responsive can be used in clinical practice to track patients’ pain-related function over time to determine additional treatment needs and to compare to population norms [10]. There are currently no plasma pain biomarkers that improve assessment and management of SCD acute or chronic pain.

Depression and anxiety as co-morbid conditions in SCD can contribute to increased pain, more pain-related distress/interference and poor coping [11]. The prevalence of depression and anxiety range from 26–33% and 6.5–36%, respectively, in adults with SCD [11–13]. Adults with SCD have an 11% higher prevalence of depression compared to Black American adults without SCD [14]. Depression and anxiety can be assessed using self-reported validated screening tools (e.g., Depression: Patient Health Questionnaire (PHQ-9) [15] for adults, Center for Epidemiologic Studies Depression Scale for Children (CES-DC) [16], PROMIS assessments for adults and children; Anxiety: Generalized Anxiety Disorder 7-item (GAD-7) scale for adults, State-Trait Anxiety Inventory for Children (STAIC) [17], PROMIS assessments for adults and children). Individuals who screen positive using these tools should be referred for evaluation by a psychologist/psychiatrist.

#### Management of acute and chronic pain

The goal of acute pain management is to provide sufficient analgesia to return patients to their usual function, which may mean complete resolution of pain for some or return to baseline chronic pain for others. The goal of chronic pain management is to optimize individuals’ function, which may not mean being pain free. When there is an identifiable cause of chronic pain, treatment of the underlying issue (e.g., joint replacement for avascular necrosis, leg ulcer treatment) is important. Opioids, oral for outpatient management and intravenous for inpatient management, are first line therapy for acute SCD pain. In the acute care setting, analgesics should be initiated within 30–60 min of triage [18]. Ketamine, a non-opioid analgesic, can be prescribed at sub-anesthetic (analgesic) intravenous doses (0.1–0.3 mg/kg per h, maximum 1 mg/kg per h) as adjuvant treatment for acute SCD pain refractory to opioids [18, 19]. In an uncontrolled observational study of 85 patients with SCD receiving ketamine infusions for acute pain, ketamine was associated with a decrease in mean opioid consumption by oral morphine equivalents (3.1 vs. 2.2 mg/kg/day, *p* < 0.001) and reductions in mean pain scores (0–10 scale) from baseline until discontinuation of the infusion (7.81 vs. 5.44, *p* < 0.001) [20]. Nonsteroidal anti-inflammatory drugs (NSAIDs) are routinely used as adjuvant therapy for acute pain treatment [18]. In a RCT (*n* = 20) of hospitalized patients with acute pain, ketorolac was associated with lower total dose of meperidine required (1866.7 ± 12.4 vs. 2804.5 ± 795.1 mg, *p* < 0.05) and shorter hospitalization (median 3.3 vs. 7.2 days, *p* = 0.027) [21]. In a case series of children treated for 70 acute pain events in the ED, 53% of events resolved with ketorolac and hydration alone with reduction in 100 mm visual analog scale (VAS) pain score from 60 to 13 (*p* < 0.001) [22]. Patients at risk for NSAID toxicity (e.g., renal impairment, on anticoagulation) should be identified.

Despite paucity of data, chronic opioid therapy (COT) can be considered after assessing benefits versus harms [23] and the functional status of patients with SCD who have chronic pain. Harms of COT seen in patient populations other than SCD are dose dependent and include myocardial infarction, bone fracture, increased risk of motor vehicle collisions, sexual dysfunction and mortality [23]. There are few published studies investigating non-opioid analgesics for chronic SCD pain [24–26]. In a randomized trial of 39 participants, those who received Vitamin D experienced a range of 6–10 pain days over 24 weeks while those who received placebo experienced 10–16 pain days, which was not significantly different [26]. In a phase 1, uncontrolled trial of 18 participants taking trifluoperazine, an antipsychotic drug, 8 participants showed a 50% reduction in the VAS (10 cm horizontal line) pain score from baseline on at least 3 assessments over 24 h without severe sedation or supplemental opioid analgesics, 7 participants showed pain reduction on 1 assessment, and the remaining 3 participants showed no reduction [24]. Although published data are not available for serotonin and norepinephrine reuptake inhibitors (SNRIs), gabapentinoids and tricyclic antidepressants (TCAs) in individuals with SCD, evidence supports their use in fibromyalgia, a chronic pain condition similar to SCD chronic pain in mechanism. A Cochrane Review that included 10 RCTs (*n* = 6038) showed that the SNRIs milnacipran and duloxetine, compared to placebo, were associated with a reduction in pain [27]. A systematic review and meta-analysis of 9 studies (*n* = 520) showed the TCA amitriptyline improved pain intensity and function [28]. Finally, a meta-analysis of 5 RCTs (*n* = 1874) of the gabapentinoid pregabalin showed a reduction in pain intensity [29]. Collectively, the indirect evidence from fibromyalgia supports the conditional recommendation in current SCD practice guidelines to consider these 3 drug classes for chronic SCD pain treatment [18]. Standard formulary dosing recommendations should be followed and reported adverse effects considered.

Non-pharmacologic therapies (e.g., integrative, psychological-based therapies) are important components of SCD pain treatment. In a case–control study of 101 children with SCD and chronic pain referred for cognitive behavioral therapy (CBT) (57 CBT, 44 no CBT) [30], CBT was associated with more rapid decrease in pain hospitalizations (estimate − 0.63, *p* < 0.05) and faster reduction in hospital days over time (estimate − 5.50, *p* < 0.05). Among 18 children who received CBT and completed PROs pre- and 12 months posttreatment, improvements were seen in mean pain intensity (5.47 vs. 3.76, *p* = 0.009; 0–10 numeric rating pain scale), functional disability (26.24 vs. 15.18, *p* < 0.001; 0–60 score range) and pain coping (8.00 vs. 9.65, *p* = 0.03; 3–15 score range) post treatment [30]. In 2 uncontrolled clinical trials, acupuncture was associated with a significant reduction in pain scores by 2.1 points (0–10 numeric pain scale) in 24 participants immediately after treatment [31] or a significant mean difference in pre-post pain scores of 0.9333 (0–10 numeric pain scale) (*p* < 0.000) after 33 acupuncture sessions [32].

### Cardiopulmonary disease

Cardiopulmonary disease is associated with increased morbidity and mortality in individuals with SCD. Pulmonary hypertension (PH), most commonly pulmonary arterial hypertension (PAH), is present based on right-heart catheterization in up to 10% of adults with SCD [33]. Chronic intravascular hemolysis represents the biggest risk factor for development of PAH in SCD and leads to pulmonary arteriole vasoconstriction and smooth muscle proliferation. Based on pulmonary function testing (PFT), obstructive lung disease may be observed in 16% of children and 8% of adults with SCD, while restrictive lung disease may be seen in up to 28% of adults and only 7% of children with SCD [34, 35]. Sleep-disordered breathing, which can manifest as obstructive sleep apnea or nocturnal hypoxemia, occurs in up to 42% of children and 46% of adults with SCD [36, 37]. Cardiopulmonary disease, including PH or restrictive lung disease, presents with dyspnea with or without exertion, chest pain, hypoxemia or exercise intolerance that is unexplained or increased from baseline. Obstructive lung disease can also present with wheezing.

#### Diagnosis of cardiopulmonary disease

The confirmation of PH in patients with SCD requires right-heart catheterization. Recently, the mean pulmonary artery pressure threshold used to define PH in the general population was lowered from ≥ 25 to ≥ 20 mm Hg [38]. Elevated peak tricuspid regurgitant jet velocity (TRJV) ≥ 2.5 m/s on Doppler echocardiogram (ECHO) is associated with early mortality in adults with SCD and may suggest elevated pulmonary artery pressures, especially when other signs of PH (e.g., right-heart strain, septal flattening) or left ventricular diastolic dysfunction, which may contribute to PH, are present [39]. However, the positive predictive value (PPV) of peak TRJV alone for identifying PH in adults with SCD is only 25% [40]. Increasing the peak TRJV threshold to at least 2.9 m/s has been shown to increase the PPV to 64%. For a peak TRJV of 2.5–2.8 m/s, an increased N-terminal pro-brain natriuretic peptide (NT-proBNP) ≥ 164.5 pg/mL or a reduced 6-min walk distance (6MWD) < 333 m can also improve the PPV to 62% with a false negative rate of 7% [33, 40, 41].

PFT, which includes spirometry and measurement of lung volumes and diffusion capacity, is standard for diagnosing obstructive and restrictive lung disease in patients with SCD. Emerging modalities include impulse oscillometry, a non-invasive method using forced sound waves to detect changes in lower airway mechanics in individuals unable to perform spirometry [42], and airway provocation studies using cold air or methacholine to reveal latent airway hyperreactivity [43]. Formal in-lab, sleep study/polysomnography remains the gold standard to evaluate for sleep-disordered breathing, which may include nocturnal hypoxemia, apnea/hypopnea events and other causes of sleep disruption. Nocturnal hypoxemia may increase red blood cell sickling, cellular adhesion and endothelial dysfunction. In 47 children with SCD, mean overnight oxygen saturation was higher in those with grade 0 compared to grade 2 or 3 cerebral arteriopathy (97 ± 1.6 vs. 93.9 ± 3.7 vs. 93.5 ± 3.0%, *p* < 0.01) on magnetic resonance angiography and lower overnight oxygen saturation was independently associated with mild, moderate or severe cerebral arteriopathy after adjusting for reticulocytosis (OR 0.50, 95% CI 0.26–0.96, *p* < 0.05) [44].

#### Management of cardiopulmonary disease

Patients with SCD who have symptoms suggestive of cardiopulmonary disease, such as worsening dyspnea, hypoxemia or reduced exercise tolerance, should be evaluated with a diagnostic ECHO and PFT. The presence of snoring, witnessed apnea, respiratory pauses or hypoxemia during sleep, daytime somnolence or nocturnal enuresis in older children and adults is sufficient for a diagnostic sleep study.

Without treatment, the mortality rate in SCD patients with PH is high compared to those without (5-year, all-cause mortality rate of 32 vs. 16%, *p* < 0.001) [33]. PAH-targeted therapies should be considered for SCD patients with PAH confirmed by right-heart catheterization. However, the only RCT (*n* = 6) in individuals with SCD and PAH confirmed by right-heart catheterization (bosentan versus placebo) was stopped early for poor accrual with no efficacy endpoints analyzed [45]. In SCD patients with elevated peak TRJV, a randomized controlled trial (*n* = 74) of sildenafil, a phosphodiesterase-5 inhibitor, was discontinued early due to increased pain events in the sildenafil versus placebo arm (35 vs. 14%, *p* = 0.029) with no treatment benefit [46]. Despite absence of clinical trial data, patients with SCD and confirmed PH should be considered for hydroxyurea or monthly red blood cell transfusions given their disease-modifying benefits. In a retrospective analysis of 13 adults with SCD and PAH, 77% of patients starting at a New York Heart Association (NYHA) functional capacity class III or IV achieved class I/II after a median of 4 exchange transfusions with improvement in median pulmonary vascular resistance (3.7 vs. 2.8 Wood units, *p* = 0.01) [47].

Approximately 28% of children with SCD have asthma, which is associated with increased pain episodes that may result from impaired oxygenation leading to sickling and vaso-occlusion as well as with acute chest syndrome and higher mortality [48–50]. First line therapies include standard beta-adrenergic bronchodilators and supplemental oxygen as needed. When corticosteroids are indicated, courses should be tapered over several days given the risk of rebound SCD pain from abrupt discontinuation. Inhaled corticosteroids such as fluticasone proprionate or beclomethasone diproprionate are reserved for patients with recurrent asthma exacerbations, but their anti-inflammatory effects and impact on preventing pain episodes in patients with SCD who do not have asthma is under investigation [51]. Finally, management of sleep-disordered breathing is tailored to findings on formal sleep study in consultation with a sleep/pulmonary specialist.

### Central nervous system (CNS) complications

CNS complications, such as overt and silent cerebral infarcts, cause significant morbidity in individuals with SCD. Eleven percent of patients with HbSS disease by age 20 years and 24% by age 45 years will have had an overt stroke [52]. Silent cerebral infarcts occur in 39% by 18 years and in > 50% by 30 years [53, 54]. Patients with either type of stroke are at increased risk of recurrent stroke [55]. Overt stroke involves large-arteries, including middle cerebral arteries and intracranial internal carotid arteries, while silent cerebral infarcts involve penetrating arteries. The pathophysiology of overt stroke includes vasculopathy, increased sickled red blood cell adherence, and hemolysis-induced endothelial activation and altered vasomotor tone [56]. Overt strokes present as weakness or paresis, dysarthria or aphasia, seizures, sensory deficits, headache or altered level of consciousness, while silent cerebral infarcts are associated with cognitive deficits, including lower IQ and impaired academic performance.

#### Diagnosis of CNS complications in SCD

Overt stroke is diagnosed by evidence of acute infarct on brain MRI diffusion-weighted imaging and focal deficit on neurologic exam. A silent cerebral infarct is defined by a brain “MRI signal abnormality at least 3 mm in one dimension and visible in 2 planes on fluid-attenuated inversion recovery (FLAIR) T2-weighted images” and no deficit on neurologic exam [57]. Since silent cerebral infarcts cannot be detected clinically, a screening baseline brain MRI is recommended in school-aged children with SCD [58]. Recent SCD clinical practice guidelines also suggest a screening brain MRI in adults with SCD to facilitate rehabilitation services, patient and family understanding of cognitive deficits and further needs assessment [58]. An MRA should be added to screening/diagnostic MRIs to evaluate for cerebral vasculopathy (e.g., moyamoya), which may increase risk for recurrent stroke or hemorrhage [59].

Annual screening for increased stroke risk by transcranial doppler (TCD) ultrasound is recommended by the American Society of Hematology for children 2–16 years old with HbSS or HbS/β° thalassemia [58]. Increased stroke risk on non-imaging TCD is indicated by abnormally elevated cerebral blood flow velocity, defined as ≥ 200 cm/s (time-averaged mean of the maximum velocity) on 2 occasions or a single velocity of > 220 cm/s in the distal internal carotid or proximal middle cerebral artery [60]. Many centers rely on imaging TCD, which results in velocities 10–15% lower than values obtained by non-imaging protocols and therefore, require adjustments to cut-offs for abnormal velocities. Data supporting stroke risk assessment using TCD are lacking for adults with SCD and standard recommendations do not exist.

Neurocognitive deficits occur in over 30% of children and adults with severe SCD [61, 62]. These occur as a result of overt and/or silent cerebral infarcts but in some patients, the etiology is unknown. The Bright Futures Guidelines for Health Supervision of Infants, Children and Adolescents or the Cognitive Assessment Toolkit for adults are commonly used tools to screen for developmental delays or neurocognitive impairment [58]. Abnormal results should prompt referral for formal neuropsychological evaluation, which directs the need for brain imaging to evaluate for silent cerebral infarcts and facilitate educational/vocational accommodations.

#### Management of CNS complications

Monthly chronic red blood cell transfusions to suppress HbS < 30% are standard of care for primary stroke prevention in children with an abnormal TCD. In an RCT of 130 children, chronic transfusions, compared to no transfusions, were associated with a difference in stroke risk of 92% (1 vs. 10 strokes, *p* < 0.001) [60]. However, children with abnormal TCD and no MRI/MRA evidence of cerebral vasculopathy can safely transition to hydroxyurea after 1 year of transfusions [63]. Lifelong transfusions to maintain HbS < 30% remain standard of care for secondary stroke prevention in individuals with overt stroke [64]. Chronic monthly red blood cell transfusions should also be considered for children with silent cerebral infarct [58]. In a randomized controlled trial (*n* = 196), monthly transfusions, compared to observation without hydroxyurea, reduced risk of overt stroke, new silent cerebral infarct or enlarging silent cerebral infarct in children with HbSS or HbS/β^0^ thalassemia and an existing silent cerebral infarct (2 vs. 4.8 events, incidence rate ratio of 0.41, 95% CI 0.12–0.99, *p* = 0.04) [57].

Acute stroke treatment requires transfusion therapy to increase cerebral oxygen delivery. Red blood cell exchange transfusion, defined as replacement of patients’ red blood cells with donor red blood cells, to rapidly reduce HbS to < 30% is the recommended treatment as simple transfusion alone is shown to have a fivefold greater relative risk (57 vs. 21% with recurrent stroke, RR = 5.0; 95% CI 1.3–18.6) of subsequent stroke compared to exchange transfusion [65]. However, a simple transfusion is often given urgently while preparing for exchange transfusion [58]. Tissue plasminogen activator (tPA) is not recommended for children with SCD who have an acute stroke since the pathophysiology of SCD stroke is less likely to be thromboembolic in origin and there is risk for harm. Since the benefits and risks of tPA in adults with SCD and overt stroke are not clear, its use depends on co-morbidities, risk factors and stroke protocols but should not delay or replace prompt transfusion therapy.

Data guiding treatment of SCD cerebral vasculopathy (e.g., moyamoya) are limited, and only nonrandomized, low-quality evidence exists for neurosurgical interventions (e.g., encephaloduroarteriosynangiosis) [66]. Consultation with a neurosurgeon to discuss surgical options in patients with moyamoya and history of stroke or transient ischemic attack should be considered [58].

### Kidney disease

Glomerulopathy, characterized by hyperfiltration leading to albuminuria, is an early asymptomatic manifestation of SCD nephropathy and worsens with age. Hyperfiltration, defined by an absolute increase in glomerular filtration rate, may be seen in 43% of children with SCD [67]. Albuminuria, defined by the presence of urine albumin ≥ 30 mg/g over 24 h, has been observed in 32% of adults with SCD [68]. Glomerulopathy results from intravascular hemolysis and endothelial dysfunction in the renal cortex. Medullary hypoperfusion and ischemia also contribute to kidney disease in SCD, causing hematuria, urine concentrating defects and distal tubular dysfunction [69]. Approximately 20–40% of adults with SCD develop chronic kidney disease (CKD) and are at risk of developing end-stage renal disease (ESRD), with rapid declines in estimated glomerular filtration rate (eGFR) > 3 mL/min/1.73 m^2^ associated with increased mortality (HR 2.4, 95% CI 1.31–4.42, *p* = 0.005) [68].

#### Diagnosis of kidney disease in SCD

The diagnosis of sickle cell nephropathy is made by detecting abnormalities such as albuminuria, hematuria or CKD rather than by distinct diagnostic criteria in SCD, which have not been developed. Traditional markers of kidney function such as serum creatinine and eGFR should be interpreted with caution in individuals with SCD because renal hyperfiltration affects their accuracy by increasing both. Practical considerations preclude directly measuring GFR by urine or plasma clearance techniques, which achieves the most accurate results. The accuracy of eGFR, however, may be improved by equations that incorporate serum cystatin C [70].

Since microalbuminuria/proteinuria precedes CKD in SCD, annual screening for urine microalbumin/protein is recommended beginning at age 10 years [71]. When evaluating urine for microalbumin concentration, samples from first morning rather than random voids are preferable to exclude orthostatic proteinuria. Recent studies suggest *HMOX1* and *APOL1* gene variants may be associated with CKD in individuals with SCD [72]. Potential novel predictors of acute kidney injury in individuals with SCD include urine biomarkers kidney injury molecule 1 (KIM-1) [73], monocyte chemotactic protein 1 (MCP-1) [74] and neutrophil gelatinase-associated lipocalin (NGAL) [75]. Their contribution to chronic kidney disease and interaction with other causes of kidney injury in SCD (e.g., inflammation, hemolysis) are not clear.

#### Management of kidney disease

Managing kidney complications in SCD should focus on mitigating risk factors for acute and chronic kidney injury such as medication toxicity, reduced kidney perfusion from hypotension and dehydration, and general disease progression, as well as early screening and treatment of microalbuminuria/proteinuria. Acute kidney injury, either an increase in serum creatinine ≥ 0.3 mg/dL or a 50% increase in serum creatinine from baseline, is associated with ketorolac use in children with SCD hospitalized for pain [76]. Increasing intravenous fluids to maintain urine output > 0.5 to 1 mL/kg/h and limiting NSAIDs and antibiotics associated with nephrotoxicity in this setting are important. Despite absence of controlled clinical trials, hydroxyurea may be associated with improvements in glomerular hyperfiltration and urine concentrating ability in children with SCD [77, 78]. Hydroxyurea is also associated with a lower prevalence (34.7 vs. 55.4%, *p* = 0.01) and likelihood of albuminuria (OR 0.28, 95% CI 0.11–0.75, *p* = 0.01) in adults with SCD after adjusting for age, angiotensin-converting enzyme inhibitor (ACE-I)/angiotensin receptor blockade (ARB) use and major disease risk factors [79].

ACE-I or ARB therapy reduces microalbuminuria in patients with SCD. In a phase 2 trial of 36 children and adults, a ≥ 25% reduction in urine albumin-to-creatinine ratio was observed in 83% (*p* < 0.0001) and 58% (*p* < 0.0001) of patients with macroalbuminuria (> 300 mg/g creatinine) and microalbuminuria (30–300 mg/g creatinine), respectively, after 6 months of treatment with losartan at a dose of 0.7 mg/kg/day (max of 50 mg) in children and 50 mg daily in adults [80]. However, ACE-I or ARB therapy has not been shown to improve kidney function or prevent CKD. Hemodialysis is associated with a 1-year mortality rate of 26.3% after starting hemodialysis and an increase risk of death in SCD patients with ESRD compared to non-SCD patients with ESRD (44.6 vs. 34.5% deaths, mortality hazard ratio of 2.8, 95% CI 2.31–3.38) [81]. Renal transplant should be considered for individuals with SCD and ESRD because of recent improvements in renal graft survival and post-transplant mortality [82].

### Disease-modifying therapies in SCD

Since publication of its landmark trial in 1995, hydroxyurea continues to represent a mainstay of disease-modifying therapy for SCD. Hydroxyurea induces fetal hemoglobin production through stress erythropoiesis, reduces inflammation, increases nitric oxide and decreases cell adhesion. The FDA approved hydroxyurea in 1998 for adults with SCD. Subsequently, hydroxyurea was FDA approved for children in 2017 to reduce the frequency pain events and need for blood transfusions in children ≥ 2 years of age [63]. The landscape of disease-modifying therapies, however, has improved with the recent FDA approval of 3 other treatments—l-glutamine and crizanlizumab for reducing acute complications (e.g., pain), and voxelotor for improving anemia (Table 3) [83–85]. Other therapies in current development focus on inducing fetal hemoglobin, reducing anti-sickling or cellular adhesion, or activating pyruvate kinase-R.

**Table 3:** Major FDA-approved therapies for the treatment of SCD

Drug and FDA approval	FDA approval date and indications	Mechanism of action	Dosing	Common adverse effects
Hydroxyurea	1998: Adults to reduce frequency of painful crises 2017: Children ≥ 2 years to reduce frequency of painful crises and need for blood transfusions	↑ Fetal Hb via temporary arrest of hematopoiesis and stress erythropoiesis ↓ Inflammation through ↓ in WBC and platelets ↓ Adhesion molecule expression ↑ Nitric oxide production	Usual starting dose 20 mg/kg/day Dose escalate to maximum tolerated dose (~ 30–35 mg/kg/day) Alternatively, dose escalate to absolute neutrophil count of 1500–2000/µL	Neutropenia (13%) Thrombocytopenia (7%) Nausea (3%)
l-glutamine	2017: Children and adults ≥ 5 years old to reduce severe complications (sickle cell crises and acute chest syndrome)	↑ NAD redox potential in sickle red blood cells Protects red blood cells from oxidative stress	Dose by weight < 30 kg—1 packet (5 g) BID 30–65 kg—2 packets (10 g) BID > 65 kg—3 packets (15 g) BID May take with or without hydroxyurea	Constipation (21%) Nausea (19%) Abdominal pain (17%) Headache (18%) Cough (16%)
Crizanlizumab	2019: Adolescents and adults ≥ 16 years old to reduce frequency of vaso-occlusive crises	Binds to P-selectin Blocks interactions with ligands, including P-selectin glycoprotein ligand 1	5 mg/kg/dose IV on weeks 0, 2 and every 4 weeks thereafter May take with or without hydroxyurea	Infusion-related adverse events (< 10%) Nausea (18%) Arthralgia (18%) Back pain (15%) Fever (11%)
Voxelotor	2019: Children and adults ≥ 12 years old to increase hemoglobin concentration	Allosteric modifier of hemoglobin to stabilize oxygenated state ↓ Sickle Hb polymerization ↓ Hemolysis	1500 mg po daily May take with or without hydroxyurea	Headache (26%) Diarrhea (20%) Nausea (17%) Abdominal pain (19%) Skin rash (14%) Fever (12%) Fatigue (14%)

#### l-glutamine

Glutamine is required for the synthesis of glutathione, nicotinamide adenine dinucleotide and arginine. The essential amino acid protects red blood cells against oxidative damage, which forms the basis for its proposed utility in SCD. The exact mechanism of benefit in SCD, however, remains unclear. In a phase 3 RCT of 230 participants (hemoglobin SS or S/β^0^ thalassemia), l-glutamine compared to placebo was associated with fewer pain events (median 3 vs. 4, *p* = 0.005) and hospitalizations for pain (median 2 vs. 3, *p* = 0.005) over the 48-week treatment period [84]. The percentage of patients who had at least 1 episode of acute chest syndrome, defined as presence of chest wall pain with fever and a new pulmonary infiltrate, was lower in the l-glutamine group (8.6 vs. 23.1%, *p* = 0.003). There were no significant between-group differences in hemoglobin, hematocrit or reticulocyte count. Common side effects of l-glutamine include GI upset (constipation, nausea, vomiting and abdominal pain) and headaches.

#### Crizanlizumab

P-selectin expression, triggered by inflammation, promotes adhesion of neutrophils, activated platelets and sickle red blood cells to the endothelial surface and to each other, which promotes vaso-occlusion in SCD. Crizanlizumab, given as a monthly intravenous infusion, is a humanized monoclonal antibody that binds P-selectin and blocks the adhesion molecule’s interaction with its ligand, P-selectin glycoprotein ligand 1. FDA approval for crizanlizumab was based on a phase 2 RCT (*n* = 198, all genotypes), in which the median rate of pain events (primary endpoint) was lower (1.63 vs. 2.68, *p* = 0.01) and time to first pain event (secondary endpoint) was longer (4.07 vs. 1.38 months, *p* = 0.001) for patients on high-dose crizanlizumab (5 mg/kg/dose) compared to placebo treated for 52 weeks (14 doses total) [83]. In this trial, patients with SCD on chronic transfusion therapy were excluded, but those on stable hydroxyurea dosing were not. Adverse events were uncommon but included headache, back pain, nausea, arthralgia and pain in the extremity.

#### Voxelotor

Polymerization of Hb S in the deoxygenated state represents the initial step in red blood cell sickling, which leads to reduced red blood cell deformability and increased hemolysis. Voxelotor is a first-in-class allosteric modifier of Hb S that increases oxygen affinity. The primary endpoint for the phase 3 RCT of voxelotor (*n* = 274, all genotypes) that led to FDA approval was an increase in hemoglobin of at least 1 g/dL after 24 weeks of treatment [85]. More participants receiving 1500 mg daily of oral voxelotor versus placebo had a hemoglobin response of at least 1 g/dL (51%, 95% CI 41–61 vs. 7%, 95% CI 1–12, p < 0.001). Approximately 2/3 of the participants in these trials were on hydroxyurea, with treatment benefits observed regardless of hydroxyurea status. Despite improvements associated with voxelotor in biomarkers of hemolysis (reticulocyte count, indirect bilirubin and lactate dehydrogenase), annualized incidence rate of vaso-occlusive crisis was not significantly different among treatment groups. Adverse events included headaches, GI symptoms, arthralgia, fatigue and rash.

### Curative therapies in SCD

For individuals with SCD undergoing hematopoietic stem cell transplantation (HSCT) using HLA-matched sibling donors and either myeloablative or reduced-intensity conditioning regimens, the five-year event-free and overall survival is high at 91% and 93%, respectively [86]. Limited availability of HLA-matched sibling donors in this population requires alternative donors or the promise of autologous strategies such as gene-based therapies (i.e. gene addition, transfer or editing) (Table 4). Matched unrelated donors have not been used routinely due to increased risk of graft-versus-host disease (GVHD) as high as 19% (95% CI 12–28) in the first 100 days for acute GVHD and 29% (95% CI 21–38) over 3 years for chronic GVHD [87]. Haplo-identical HSCT, using biological parents or siblings as donors, that incorporate post-transplant cyclophosphamide demonstrates acceptable engraftment rates, transplant-related morbidity and overall mortality [88]. Regardless of allogeneic HSCT type, older age is associated with lower event-free (102/418 vs. 72/491 events, HR 1.74, 95% CI 1.24–2.45) and overall survival (54/418 vs. 22/491 events, HR 3.15, 95% CI 1.86–5.34) in patients ≥ 13 years old compared to < 12 years old undergoing HSCT [87].

**Table 4:** Therapies with curative intent

*Allogeneic hematopoietic stem cell transplant*	
HLA-matched sibling donor	Standard approaches rely on myeloablative conditioning with overall and disease-free survival rates in the 90% range for children and young adults with SCD Reduced intensity and reduced toxicity, nonmyeloablative regimens offer alternative strategies to achieve stable, mixed chimerism in adults with SCD
Umbilical cord donor	Cord units from related or unrelated donors represent alternative sources of hematopoietic stem cells for children with SCD; routine use is limited by lower total cell dose Units from unrelated donors are associated with higher risk of graft rejection and graft-versus-host disease
HLA-matched unrelated donor	Demographics of current donor pool limit this option for Black patients with SCD High rates of acute and chronic graft-versus-host disease remain a significant challenge
Haploidentical donor	Haploidentical donors offer most accessible donor type for children and adults with SCD Strategies focused on T-cell depletion using post-transplant cyclophosphamide have improved engraftment rates and reduced graft-versus-host disease
*Autologous gene-based therapy*	
Gene addition or transfer	Lentiviral-based vector encodes modified β- or γ-globin transgenes to increase anti-sickling hemoglobin production Lentiviral-based vector transfers short-hairpin RNA (shRNA) targeting *BCL11A* to increase γ-globin expression Transduction efficiency is high with current vectors Concerns remain about risk of insertional oncogenesis and long-term high-level expression
Gene editing	Genome editing focuses on correction of SCD mutation, downregulation of *BCL11A* or mimicking of hereditary persistence of fetal hemoglobin (HPFH) mutations Strategies rely on CRISPR-Cas9 technology or engineered DNA-cleaving enzymes such as zinc-finger nucleases (ZFNs) or transcription activator-like effector nucleases (TALENs)

### Advancing research in SCD

Despite progress to date, additional high-quality, longitudinal data are needed to better understand the natural history of the disease and to inform optimal screening for SCD-related complications. In the era of multiple FDA-approved therapies with disease-modifying potential, clinical trials to evaluate additional indications and test them in combination with or compared to each other are needed. Dissemination and implementation studies are also needed to identify barriers and facilitators related to treatment in everyday life, which can be incorporated into decision aids and treatment algorithms for patients and their providers [89]. Lastly, continued efforts should acknowledge social determinants of health and other factors that affect access and disease-related outcomes such as the role of third-party payers, provider and patient education, health literacy and patient trust. Establishing evidence-derived quality of care metrics can also drive public policy changes required to ensure care optimization for this population.

## Conclusions

SCD is associated with complications that include acute and chronic pain as well as end-organ damage such as cardiopulmonary, cerebrovascular and kidney disease that result in increased morbidity and mortality. Several well-designed clinical trials have resulted in key advances in management of SCD in the past decade. Data from these trials have led to FDA approval of 3 new drugs, l-glutamine, crizanlizumab and voxelotor, which prevent acute pain and improve chronic anemia. Moderate to high-quality data support recommendations for managing SCD cerebrovascular disease and early kidney disease. However, further research is needed to determine the best treatment for chronic pain and cardiopulmonary disease in SCD. Comparative effectiveness research, dissemination and implementation studies and a continued focus on social determinants of health are also essential.

## Data Availability

Not applicable.

## Abbreviations

6-MWD: Six-minute walk distance
ACE-I: Angiotensin-converting enzyme inhibitor
ARB: Angiotensin receptor blockade
CBT: Cognitive behavioral therapy
CKD: Chronic kidney disease
COT: Chronic opioid therapy
ECHO: Echocardiogram
ESRD: End stage renal disease
FLAIR: Fluid-attenuated inversion recovery
GFR: Glomerular filtration rate
GVHD: Graft-versus-host disease
HbS: Hemoglobin S
HSCT: Hematopoietic stem cell transplant
NSAIDs: Nonsteroidal anti-inflammatory drugs
NT-proBNP: N-terminal pro-brain natriuretic peptide
NYHA: New York Heart Association
PAH: Pulmonary arterial hypertension
PFT: Pulmonary function test
PH: Pulmonary hypertension
PPV: Positive predictive value
PROs: Patient-reported outcomes
RCT: Randomized controlled trial
SCD: Sickle cell disease
SNRIs: Serotonin and norepinephrine reuptake inhibitors
TCAs: Tricyclic antidepressants
TCD: Transcranial Doppler
tPA: Tissue plasminogen activator
TRJV: Tricuspid regurgitant jet velocity
VAS: Visual Analog Scale
